# Influence of high altitude on the expression of HIF-1 and on the prognosis of Ecuadorian patients with gastric adenocarcinoma

**DOI:** 10.18632/oncotarget.28275

**Published:** 2022-09-14

**Authors:** Edwin Cevallos Barrera, Edson Zangiacomi Martinez, Mariangela Ottoboni Brunaldi, Eduardo Antonio Donadi, Ajith Kumar Sankarankutty, Rafael Kemp, José Sebastiao dos Santos

**Affiliations:** ^1^Universidad Central del Ecuador, Ciencias Médicas, Carrera de Medicina, Hospital de Especialidades de Fuerzas Armadas HE-1, Quito, Ecuador; ^2^Department of Surgery and Anatomy, Ribeirao Preto Medical School, University of São Paulo, São Paulo, Brazil; ^3^Department of Social Medicine, Ribeirao Preto Medical School, University of São Paulo, São Paulo, Brazil; ^4^Department of Pathology, Ribeirao Preto Medical School, University of São Paulo, São Paulo, Brazil; ^5^Department of Internal Medicine, Ribeirao Preto Medical School, University of São Paulo, São Paulo, Brazil

**Keywords:** gastric adenocarcinoma, hypoxia-induced factor, HER2, survival rate, tumor markers

## Abstract

Since the incidence of gastric adenocarcinoma (GA) is high in populations living at high altitudes, we evaluated the influence of altitude on the expression of HIF-1 and survival of Ecuadorian GA patients. Method: 155 GA cases were studied: 56 from coastal (GAC) and 99 from mountainous regions (GAM), and 74 non-GA controls (25 coast and 49 mountain). The expression of HIF-1/HER2 was analyzed by immunohistochemistry. Analyses were performed using Fisher's exact and Breslow-Day tests for homogeneity and Kaplan-Meier curves and restricted median survival time ΔRMST. Results: HIF-1 was overexpressed in normal/inflamed gastric mucosa, especially in mountainous non-GA patients (*p* = 0.001). There was no difference between GAC and GAM in terms of age/gender, HIF-1/HER2 expression, stage/tumor location. Median survival at 120 months was significantly higher among GAC, with a difference (ΔRMST) of 43.7 months (95% CI 29.5, 57.8) (*p* < 0.001) and those with positive HIF-1 expression: ΔRMST 26.6 months (95% CI 11.0, 42.1) (*p* < 0.001). Positive HIF-1 expression was associated with better GAM survival, with ΔRMST 33.6 months (95% CI 14.2, 52.9) (*p* < 0.001). Conclusion: Despite the limitations of this retrospective study, GA patients in the coastal region and those who expressed HIF-1 exhibited a better prognosis, but this factor was associated with better survival only in the mountain region.

## INTRODUCTION

Cancer incidence and mortality are growing rapidly around the world, becoming a major barrier to increasing life expectancy [1]. Gastric cancer is the third leading cause of death in the world and is estimated to cause almost 15 million deaths by 2035 [2]. Gastric adenocarcinoma (GA) has a high incidence in Ecuador, in men it ranks third and in women it ranks fifth.

Ecuador has a varied altitude diversity and there is a differential incidence of cancer between populations living in the Andean or mountainous region when compared to coastal populations or living at low altitude. In Quito and Ambato, cities located at 2800 meters above sea level, GA also has a very high incidence: 19.6 in men and 13.9 in women per 100,000 inhabitants. There is also a high mortality rate: 16.3 in men and 11.0 in women per 100,000 inhabitants [3, 4]. In Guayaquil, located 100 meters above sea level, the incidence rate of GA is 7.5 in men and 5.2 in women per 100,000 inhabitants, while mortality is 6.20 deaths in 2009; 5.91 deaths in 2013, and 6.32 in 2018 per 100,000 inhabitants [5].

Several factors may act as promoters of gastric cancer including dietary, environmental, and demographic factors; however, there is a broad description of the genetic and molecular factors associated with carcinogenesis and response to the treatment. The TOGA study showed that HER2+ patients, who received chemotherapy plus Trastuzumab, had higher response rates and longer overall survival. It is likely that other receptors, such as HER3 and HER4, also have a similar potential response to other monoclonal antibodies [6].

The association between geographic altitude, regulation of gene expression sensitive to changes in oxygen levels, tissue hypoxia factors and cancer has been the subject of multiple studies [7]. The expression of hypoxia-inducing factors (HIF), deficiency of vitamin D, increased ultraviolet radiation, oxygen toxicity and physiological changes in pH have been implicated on altitude-induced life damage. [8, 9] Cells subjected to hypoxia could favor the expression of HIF genes responsible for cell proliferation, angiogenesis, metastasis, and even increased metabolism [10].

In gastric cancer patients, HIF-1 activation after hypoxia strongly correlates with an aggressive tumor phenotype and poor prognosis. HIF-1 activation has also been reported to occur through mechanisms independent of hypoxia, such as PI3K/AKT/mTOR signaling and ROS production. Critical roles of HIF-1 are found in glucose metabolism, carcinogenesis, angiogenesis, invasion, metastasis, cell survival, and chemo resistance of gastric cancer [8, 11, 12]. Previous studies have also reported that HIF-1 is involved in resistance to anti-cancer chemotherapy drugs [13].

Considering that: I) altitude may influence the expression of HIF-1 in GA patients, II) GA is very frequent in Ecuador particularly in areas of high altitudes (Quito and Ambato, both situated 2800 meters above sea level) when compared to low-altitude areas (Guayaquil, situated 100 meters above sea level), we evaluated HIF-1 expression and its impact on patient survival in these distinct Ecuadorian areas.

## RESULTS

We analysed the medical records of 674 patients from two oncology hospitals and a specialty hospital in the Ecuadorian mountainous region and one oncology hospital in the coastal region. Among these medical records, 438 did not contain complete information on the variables considered essential for the study and only 229 patients met the inclusion criteria (56 GA from coastal and 99 GA from the mountainous areas, and 25 non-GA from the coastal and 49 non-GA from the mountainous regions), as shown in Table 1.

**Table 1:** Demographic, clinicopathological, and treatment features of Ecuadorian patients exhibiting gastric adenocarcinoma from the coastal or mountainous regions

Variable		Costal (*n*)	Mountain (*n*)	Total	*p* value^a^
**Age**	60 years	30 (53.6)	51 (51.5)	81	0.868
	>60 years	26 (46.4)	48 (48.5)	74	
**Gender**	Women	27 (48.2)	37 (37.4)	64	0.235
	Men	29 (51.8)	62 (62.6)	91	
**Histology pattern**	Intestinal	29 (51.8)	58 (58.6)	87	0.011
	Diffuse	22 (39.3)	41 (41.4)	63	
	Mixed	5 (8.9)	0 (0.0)	5	
**Tumor stage (*n* = 155)**	IAIB	4 (7.1)	6 (6.1)	10	0.275
	II	8 (14.3)	25 (25.3)	33	
	IIIA	7 (12.5)	15 (15.2)	22	
	IIIB	13 (23.2)	19 (19.2)	32	
	IVM0	10 (17.9)	7 (7.1)	17	
	IVM1	14 (25.0)	27 (27.3)	41	
**Tumor location**	cardia	3 (5.4)	6 (6.1)	9	1.000
	body	53 (94.6)	93 (93.9)	146	
**Treatment**	surgery and/or chemo/radiotherapy	56 (32.1)	83 (16.2)	139	0.004
	chemotherapy radiotherapy of palliative care	0 (0.0)	16 (1.0)	16	

^a^Fisher’s Exact test.

Since the major goal of the study was the evaluation of HIF-1 expression, the control group was primarily included to observe differences regarding this marker. As expected, HER2 expression was not observed in the control group.

Considering the stratification of GA patients according the regions, the intestinal Lauren histologic type was more frequent than the diffuse type in both regions. Regarding treatment, surgery alone or combined with chemotherapy/radiotherapy was more frequently performed in the coastal area (*p* = 0.004).

### Expression of HIF-1

The comparisons of GA patients with controls, stratified by region, showed that HIF-1 expression was identified in: I) 37 patients (66.1%) of the coastal region and in 8 controls (32.0%) (*p* = 0.0070), and II) 43 GA patients (43.4%) of the mountainous region and in 47 controls (95.9%) (*p* > 0.001) Table 2.

**Table 2:** Expression of HIF-1 in gastric adenocarcinoma patients, stratified according to geographical provenance regions; i.e., mountainous or coastal areas, and respective controls

	Coast					Montainous					Coast × Montainous
	Adeno carcinoma	Control	Total	OR (95%CI)	*p* value^a^	Adeno carcinoma	Control	Total	OR (95%CI)	*p* value^a^	*p* value^b^
**Age**											
≤ 60 years	30 (53.6)	13 (52.0)	**43**	0.94 (0.43–2.04)	1.000	51 (51.5)	25 (51.0)	**76**	0.98 (0.53–1.82)	1.000	0.942
>60 years	26 (46.4)	12 (48.0)	**38**			48 (48.5)	24 (49.0)	**72**			
**Gender**											
Woman	27 (48.2)	18 (72.0)	**45**	2.76 (1.12–6.67)	0.056	37 (37.4)	32 (65.3)	**69**	3.15 (1.61–6.25)	0.002	0.834
Men	29 (51.8)	7 (28.0)	**36**			62 (62.6)	17 (34.7)	**79**			
**Expression of HIF-1**											
Negative	19 (33.9)	17 (68.0)	**36**	4.14 (1.69–10.0)	0.007	56 (56.6)	2 (4.1)	**58**	0.03 (0.01–0.10)	<0.001	<0.001
Positive	37 (66.1)	8 (32.0)	**45**			43 (43.4)	47 (95.9)	**90**			

^a^Fisher's exact test. ^b^Homogeneity test (Breslow-Day).

**Figure 1:**
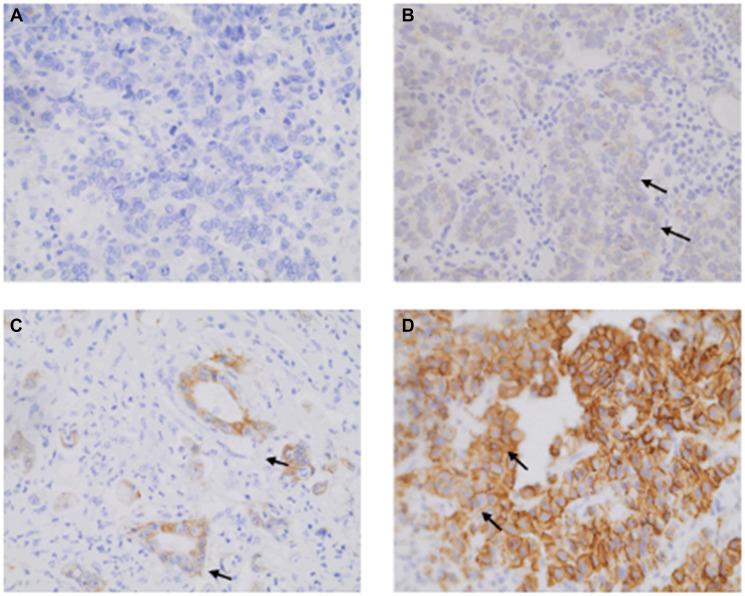
Illustrative HER2 immunohistochemistry labeling observed for gastric adenocarcinoma (GA) patients at 400× magnification. (**A**) Lack of labelling (score 0), (**B**) Weak labelling (arrows; score 1), (**C**) Weak to moderate staining (score 2), and (**D**) Strong membrane labelling (arrows; score 3).

**Figure 2:**
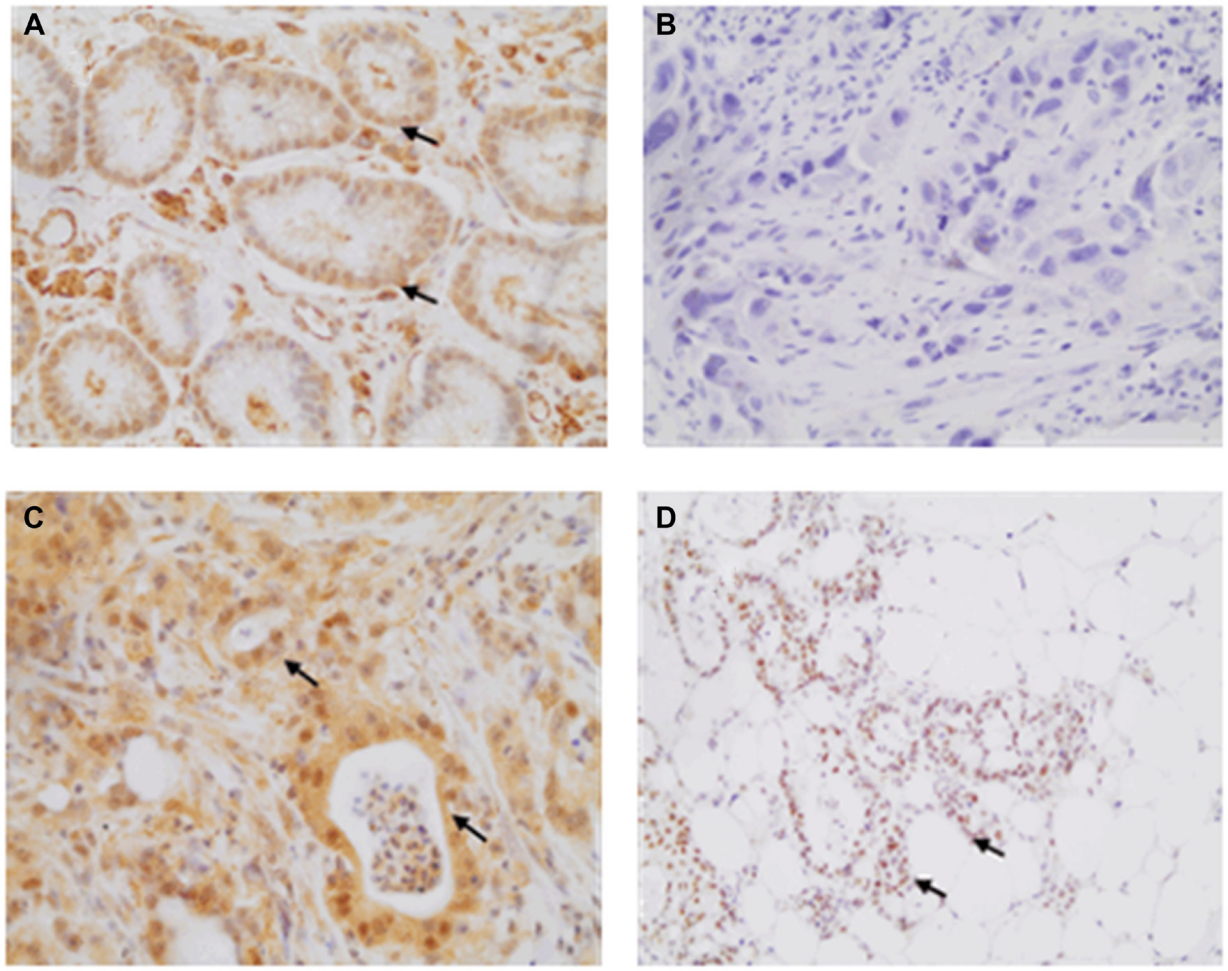
Illustrative HIF-1 immunohistochemistry labeling observed at 400× magnification for gastric samples. (**A**) Gastric mucosa without gastric adenocarcinoma (GA) (score 8), (**B**) Lack of labelling in a GA patient (score 0), (**C**) Moderate expression observed in more than 75% of the nuclei (score 8), and (**D**) Strong labelling observed in more than 75% of the nuclei (score 12).

The statistical analyses referring to the HER2 and HIF-1 expression and the expression patterns of these markers are shown in Table 3 and Figures 1 and 2.

**Table 3:** Demographic features of patients exhibiting gastric adenocarcinoma (GA) and controls individuals, exhibiting gastritis or normal mucosa

Variable		GA	Controls	Total	OR (95% CI)	*p* value^a^
Age	≤ 60 years	81 (52.3%)	38 (51.4%)	119	0.96 (0.55–1.69)	1.000
	> 60 years	74 (47.7%)	36 (48.6%)	110		
Region	Coastal	56 (36.1%)	25 (33.8%)	81	0.90 (0.51–1.59)	0.769
	Mountainous	99 (63.9%)	49 (66.2%)	148		
Gender	Woman	64 (41.3%)	50 (67.6%)	114	2.97 (1.64–5.26)	<0.001
	Men	91 (58.7%)	24 (32.4%)	115		
HIF-1 expression (all individuals)	Negative	75 (48.4%)	19 (25.7%)	94	0.37 (0.20–0.66)	0.001
	Positive	80 (51.6%)	55 (74.3%)	135		
HIF-1 expression in GA patients stratified by altitude regions	Coastal	37 (66.1%)	8 (80.4%)	45	NA	<0.001
	Mountainous	43 (43.4%)	47 (95.9%)	90		
Expression of HER-2	Negative	141 (91.0%)	74 (100.0%)	215	NC	0.006
	Positive	14 (9.0%)	0 (0.0%)	14		

^a^Fisher’s Exact Test. Abbreviations: NC: Not calculated; ND: Non available data. The statistical analyses (Odds ratio, 95% confidence interval, and *p* values) regarding the comparisons of HIF-1 and HER2 immunohistochemical expression in GA patients (whole group or stratified according to coastal or mountainous regions) with controls are also shown.

### Survival rate

**Figure 3:**
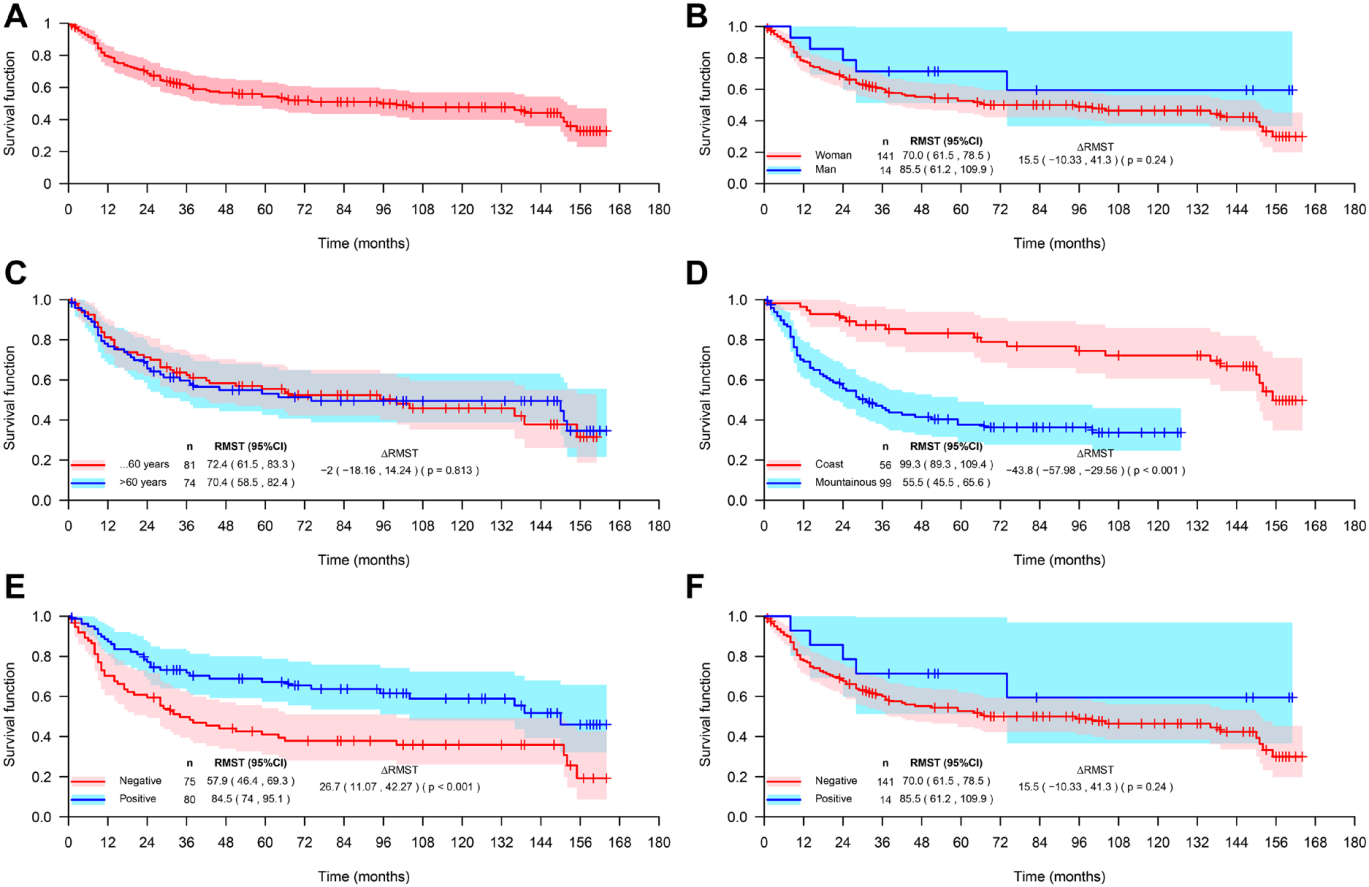
Overall survival of gastric adenocarcinoma (GA) patients. (**A**) All studied patients (*n* = 155), and of GA patients stratified by gender (**B**), age (**C**), coastal or mountainous regions (**D**), expression of HIF-1 (**E**), and expression of HER2 (**F**).

**Figure 4:**
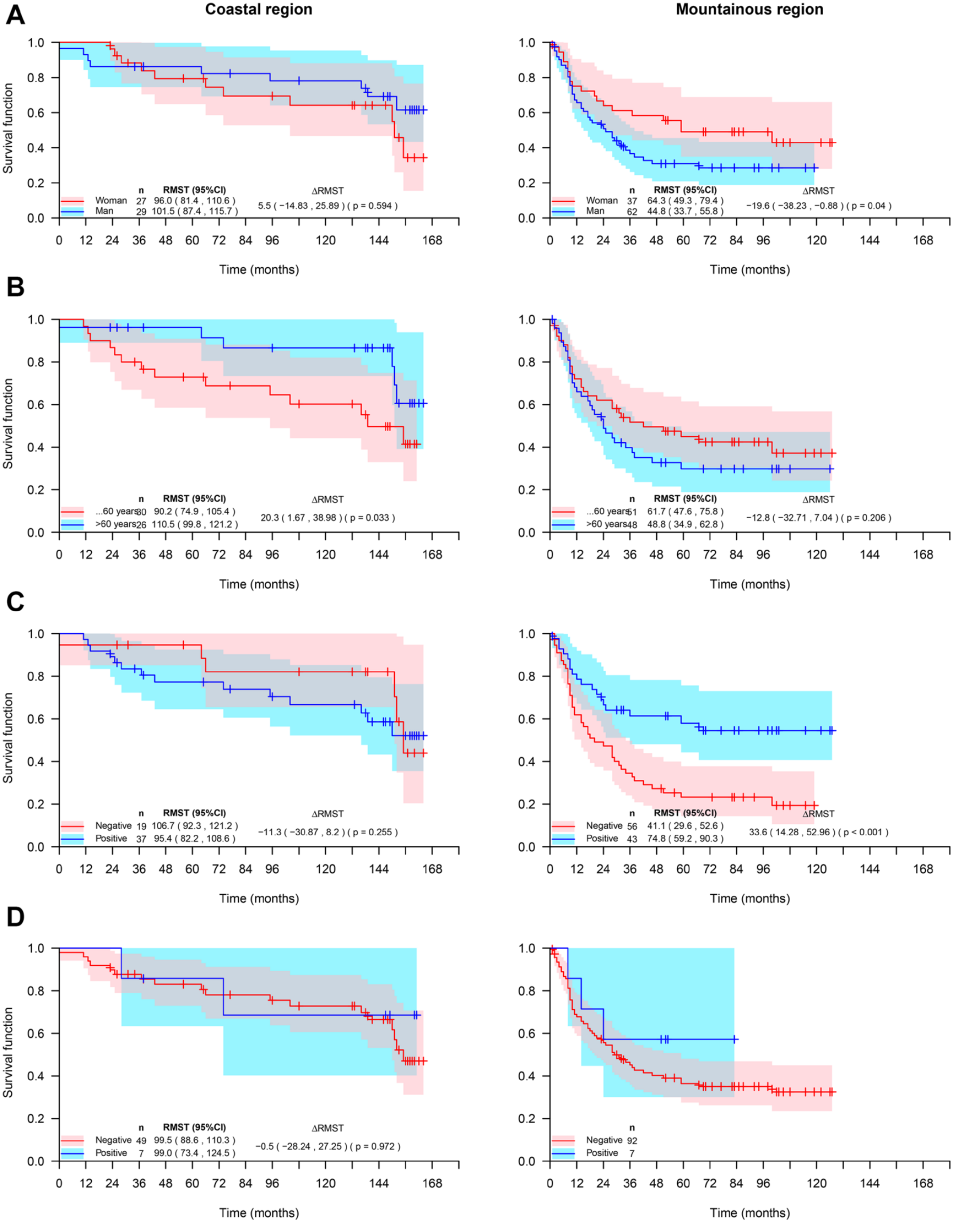
Survival rate of Ecuadorian gastric adenocarcinoma patients from coastal or mountainous regions, stratified by gender (**A**), Age (**B**), Expression of HIF-1 (**C**), and expression of HER2 (**D**).

The overall survival of patients with GA was not influenced by gender, age groups (more or less than 60 years old) and HER2 expression, however, patients from the coastal region and with positive expression of HIF-1 had better survival (Figure 3, Table 4). The survival rate of GA coastal area patients was positively influenced by the age (over 60 years), whereas in mountainous GA patients the survival rate was positively influenced by the female gender and by the expression HIF-1 (Figure 4 and Table 4).

**Table 4:** Survival rate stratified by gender, age, expression of HIF-1 and HER2 in gastric adenocarcinoma patients of mountainous and coast region showing the RMST and ΔRMST, (when needed), values at 24, 60, 90 and 120 months

Variable	Coast			Mountainous		
	RMST (95%CI) 24 month	RMST (95%CI) 60 month	RMST (95%CI) 120 month	RMST (95%CI) 24 month	RMST (95%CI) 60 month	RMST (95%CI) 120 month
**Gender**						
Woman	24.0 (23.9, 24.0)	54.3 (49.7, 58.9)	96.2 (81.7, 110.7)	19.4 (17, 21.8)	40.6 (33.1, 48.2)	68.6 (52.2, 85)
Men	22.1 (20.2, 24.0)	53.1 (46.8, 59.4)	101.7 (87.6, 115.8)	17.4 (15.5, 19.4)	30.6 (25.0, 36.2)	47.7 (35.6, 59.8)
ΔRMST	1.9 (0, 3.8)	1.2 (−6.6, 9.0)	−5.5 (−25.7, 14.7)	2.0 (−1.1, 5.1)	10.0 (0.6, 19.4)^#^	20.9 (0.5, 41.2)^#^
**Age**						
≤60 years	22.9 (21.8, 24.1)	50.5 (44.6, 56.5)	90.4 (75.2, 105.6)	18.5 (16.4, 20.7)	37.3 (30.8, 43.9)	49.2 (35.3, 63.1)
>60 years	23.1 (21.4, 24.8)	57.7 (53.3, 62.1)	110.6 (99.9, 121.2)	17.8 (15.6, 20.0)	31.3 (24.9, 37.7)	61.9 (47.9, 76.0)
ΔRMST	−0.2 (−2.3, 1.9)	−7.2 (−14.5, 0.2)	−20.2 (−38.7, −1.6)^#^	0.7 (−2.3, 3.8)	6.0 (−3.1, 15.2)	12.8 (−7.0, 32.5)
**HIF−1**						
Negative	22.8 (20.4, 25.1)	56.9 (50.9, 62.8)	106.8 (92.5, 121.2)	16.8 (14.7, 18.9)	28.5 (22.8, 34.2)	41.5 (30.1, 53.0)
Positive	23.1 (22.2, 24.1)	52.2 (47.2, 57.2)	95.6 (82.5, 108.7)	20.0 (17.9, 22.1)	42.5 (35.6, 49.4)	75.1 (59.6, 90.7)
ΔRMST	−0.3 (−2.9, 2.2)	4.7 (−3.1, 12.5)	11.2 (−8.1, 30.6)	−3.3 (−6.2, −0.3)^#^	−14.0 (−22.9, −5.0)^#^	−33.6 (−52.9, −14.2)^#^
Adj. ΔRMST (a)	−0.8 (−3.4, 1.7)	3.5 (−4.5, 11.6)	9.4 (−11.3, 30.3)	−3.1 (−6.0, −0.1)^#^	−12.7 (−21.6, −3.9)^#^	−20.9 (−41.0, −0.9)^#^
**HER2**						
Negative	22.9 (21.7, 24.0)	53.6 (49.3, 57.9)	99.6 (88.8, 110.4)	18.0 (16.4, 19.6)	33.9 (29.1, 38.7)	(b)
Positive	24.0 (24.0, 24.0)	55.5 (47.4, 63.6)	99.1 (73.8, 124.5)	20.4 (16.1, 24.8)	41.1 (24.6, 57.6)	(b)
ΔRMST	−1.1 (−2.3, 0)	−2.0 (−11.1, 7.2)	0.5 (−27.1, 28.0)	−2.4 (−7.1, 2.2)	−7.2 (−24.3, 10.0)	(b)
Adj. ΔRMST (a)	(b)	−2.7 (−12.1, 6.7)	−2.9 (−32.1, 26.4)	−2.4 (−6.7, 1.9)	−8.5 (−27.3, 10.2)	(b)

^#^p < 0.05, (a) ΔRMST adjusted by gender and age, (b) It was not possible to estimate.

## DISCUSSION

The present study is opportune to verify the relationship between altitude hypoxia and GA, taking advantage of the real circumstances of treatment for patients who reside in the coastal region at 100 meters from sea level and those who reside in the mountainous region at approximately 2,800 meters from sea level.

All patients were followed-up and treated in hospital services belonging to the same institution, the Society for Combating Cancer (SOLCA) of the Ecuador. Of the 155 GA cases evaluated, 64 (41.3%) were women and 91 (58.7%) men, and this male predominance is similar to that observed in epidemiological studies [14]. In addition, the predominance of men was higher in the sample of GA cases attended in the mountain (62.6%), while in the coast it was 51.8%, without reaching significance. These findings may represent a bias in the study design and exclusion criteria, such as loss of follow-up, which is more frequent among men, since in Guayaquil (coastal area) the men/women ratio is 1.7 [5].

Notwithstanding the gender difference in the coastal region, the GA survival rate was not affected by gender in this area. In contrast, in the mountainous population women had a better survival compared to men. Overall, the increased female survival rate for GA patients is in agreement with previous reports [15].

Patients of the present study exhibited an average age of 60 years in the coastal region and of 59 years in the mountain region. The survival rate for patients with gastric cancer is greater in patients over 60 years [16]. Indeed, in the coastal area of Ecuador the survival rate for GA patients aged over 60 years was greater than those under 60 years. In contrast, in the mountainous region the survival rate for patients under 60 years was greater than that observed for patients over 60 years, indicating the mountainous GA patients may exhibit additional risk factors. It is noteworthy that there is no difference in the GA staging of the cases evaluated between the two regions, but in the coastal area the number of tumor resections was greater than for patients of the mountainous area, and the number of palliative care patients was greater in the mountain region.

In the present study, the HER2 expression in GA patients was only 7% both on the coastal and in the mountainous areas, and was not expressed in patients without adenocarcinoma. HER2 expression has been primarily associated with the intestinal subtype [17], a result that was not observed in the present study.

A recent meta-analysis showed that in 7 out of 15 papers the HER2 positivity was associated with a worse prognosis. In the present study, we observed no significant differences between the survival rate and the HER2 expression in both groups of GA patients, even after adjusting the RMST values for age and gender.

The major focus of the present study was the investigation of the role of HIF-1 in the pathogenesis and prognosis of GA patients, taking advantage of having access to Ecuadorian oncological services from coastal and mountainous regions, where patients are submitted to differential degrees of chronic hypoxia.

To study the differential HIF-1 expression in gastric samples, we evaluated GA samples from high and low altitudes together with their respective non-GA samples, which served as controls. In short, considering only the non-GA samples, HIF-1 was observed in 32% of the coastal samples and in 95.9% of the mountainous samples. Indeed, we observed the expression of HIF-1 in individuals exhibiting normal biopsies, a finding that differs from other studies, reporting no or low HIF-1 in normal tissue [18, 19]. In contrast to non-GA, HIF-1 was observed in 66.1% of the coastal and in 43.4% of the mountainous GA samples.

Although the control group exhibited gastritis as well as normal mucosa, the overexpression of HIF-1 in mountainous regions compared to the coastal regions indicates that the chronic hypoxia induces the expression of HIF-1, which may be an adaptation of the gastric mucosa to high altitudes, “protecting” the gastric mucosa. Therefore, HIF-1 expression appears to be an early and significant event in normal gastric mucosa or in gastritis, without *H. pylori* infection in patients living in the high-altitude regions.

Noteworthy, compared to controls, mountainous GA patients exhibited an under expression of HIF-1, further indicating that the loss of HIF-1 expression may be detrimental for patients living in high altitudes. The finding that GA mountainous patients exhibiting HIF-1 expression presented increased survival rate when compared to those who did not express HIF-1, even after adjusting the RMST values by gender and age, corroborates this idea.

If, by one hand, hypoxia is a common feature for solid tumors, contributing locally and systemically to tumor progression, lack of response to radiotherapy and chemotherapy, increasing the probability of tumor recurrence [20], on the other hand, the transcription factor HIF-1 that is the major regulator of tumor adaptation to hypoxia, may induce the expression of an array of genes with distinct functions [21]. Therefore, the final effect of HIF-1 may depend on the myriad of protein/protein, protein/DNA interactions within the cell nucleus, among other risk factors. In the context of gastric cancer, HIF-1 is one among a large group of risk factors associated with the GA pathogenesis and prognosis.

Concluding, this study suggests that HIF-1 has a differential expression pattern in gastric samples according to geographical features, being highly expressed even in non-carcinomatous cells (gastritis and normal mucosa) from individuals living in regions of high altitude, indicating that the gastric HIF-1 expression may be an adaptation of the individual to high altitudes. Considering that: I) the HIF-1 expression in GA was underrepresented in high altitude areas when compared to controls, and II) the HIF-1 expression in GA samples from high altitudes was associated with better survival rate when compared to samples that do not express HIF-1, irrespective of the age and gender of patients, one may hypothesize that the expression HIF-1 is a protective marker for this population. Finally, studies evaluating the HIF-1 expression should consider altitude as a confusion factor for understanding the role of HIF-1 on GA pathogenesis and prognosis.

## MATERIALS AND METHODS

This is an observational, case-control, cross-sectional, retrospective study. Data were obtained from the electronic medical records of patients diagnosed with GA, and all clinical investigations were carried out in accordance with the principles outlined in the Declaration of Helsinki. The study protocols were approved by the following Ethics Research Committees on Human Beings: I) SOLCA of Quito, II) Central University of Ecuador, III) Hospital of the Unit Oncology of Tungurahua, Ecuador, IV) SOLCA, Guayaquil Hospital, and V) University Hospital of the Ribeirao Preto Medical School, Brazil.

### Study population

We studied 229 individuals, 155 GA cases 99 from the mountainous region of Quito and Ambato and 56 from the Guayaquil coastal region, followed-up in four hospitals from 2005 to 2018. Most of the patients underwent gastrectomy and some of them underwent adjuvant treatment; however, some patients did not accept treatment. No patient underwent neoadjuvant chemotherapy or radiotherapy. Patient inclusion criteria encompassed: I) histopathological diagnosis of GA, II) comprehensive management in one of the SOLCA oncology hospitals (Quito, Ambato, Guayaquil), and III) the availability of blocks of paraffin processed with buffered formaldehyde for the immunohistochemical studies. Exclusion criteria included: I) patient management or partial management outside SOLCA cancer hospitals, II) patient death in the staging phase or initial treatment, III) patients who were not treated in these cancer centers, and IV) the presence of histologic gastric cancer features other than GA.

In the absence of symptoms, physical examination was performed every 3–6 months during survival time. Follow-up consisted of clinical evaluation, complete blood count, liver function tests, lung, abdominal, and pelvic CT. The date of the first relapse and the date of death were recorded, survival was calculated from the time of histopathological diagnosis, until the last follow-up or death from any cause.

In parallel, we studied 74 patients (49 patients from Quito and Ambato and 25 patients from Guayaquil) without GA, most of them (89%) exhibited gastritis and the remaining exhibited normal mucosa, and none of the controls exhibited *Helicobacter pylori*. Demographic features of the studied individuals are shown in Table 1.

### Histological analyses

GA samples were obtained from endoscopic biopsies or surgical resection, and stained with the hematoxylin and eosin. Tumors were classified according to Lauren, defining the intestinal, diffuse, mixed and indeterminate types. Diffuse carcinomas consisted of loosely cohesive neoplastic cells with little or no glandular formation. Intestinal carcinomas were formed by glands of different degrees of differentiation. Tumors composed of intestinal and diffuse components in equivalent proportions were called mixed. Undifferentiated tumors were classified as indeterminate [14, 15].

### Immunohistochemical analyses for HER2 and HIF1α

The GA samples previously prepared at the SOLCA hospitals were submitted to deparaffinization, diaphanization and hydration. Antigen retrieval was carried out using hot moist, in the Optisteam Plus steam cooker with the high pH buffer (EnVision Kit, Agilent, Santa Clara, CA), for 45 minutes. Endogenous peroxidase was blocked with 3% hydrogen peroxide in PBS pH 7.2 buffer. Slides were incubated for one hour with primary antibodies (rabbit polyclonal antihuman C-erbB-2-oncoprotein, diluted 1:500: DAKO code A-0485, Glostrup, Denmark, and rabbit polyclonal against HIF-1 alpha, dilutes 1:175: Abcam ab82832, Cambridge, UK). After incubation, three consecutive washes with TBST buffer were performed, followed by incubation in the EnVision Flex HRP-Dako detection system (code K8002) for 30 minutes. Then, three consecutive washes were performed using TBST buffer. The reaction was run with diaminobenzidine (DAB) from the EnVision Kit (K8002) in 5 minutes. Positive controls were those recommended by the manufacturer for each antibody. Negative controls were obtained using the same histological sections used in the positive controls, replacing the primary antibody with diluent.

### Labelling evaluation

All immunohistochemical evaluations were performed by experienced pathologists who were unaware of the study groups.

Criteria for interpreting HER2 immunohistochemical findings in GA included a full membrane, basolateral, or lateral labelling pattern. Samples exhibiting weak/moderate and strong labelling in ≥10% of the cells in at least 5 pooled tumour cells were considered positive (scores 2 and 3). Samples with lack of reactivity or membranous reactivity in <10% of tumour cells (score 0) or weak or barely perceptible membrane reactivity in <10% of tumour cells in surgical specimens and in groups of at least 5 pooled tumour cells (score 1) were considered negative [16, 17] (Table 1).

Criteria for the immunohistochemical analysis of HIF-1 included the evaluation of nuclear staining, as defined by the intensity of the staining and the percentage of positive nuclei. A colour intensity value of 0 was classified as negative; 1 represented a weak staining; 2 as moderate; and 3 as strong staining. The percentage of nuclear positivity was stratified into: 0- negative; 1- positive in ≤10% of cells; 2- positive in >10% and ≤50% of cells; 3- positive in >50% and ≤75% of cells; and 4- positive in >75% of cells. The two scores were multiplied and expressed as: 0 negative; 1–4 weak expression; 5–8, moderate expression; and 9–12, strong expression. The mean score was used as a cut off point for expression (mean score = 6). Finally, samples were classified into the following groups: negative - no expression, low expression (scores 1–6), and high expression (scores 7–12) [18–20]. Table 2 shows these results.

### Statistical analyses

Associations between demographic and clinicopathological features and tumour marker expression were performed using Fisher’s exact test. Kaplan-Meier curves were used to analyse overall survival according to classes of variables of interest (gender, age, altitude region, and HIF-1 and HER2 expression). After checking for proportionality of hazards, we verified that standard Cox proportional hazards models and the hazard ratio (HR) measures were inappropriate for our data. Alternatively, restricted median survival time (RMST) was used as a summary statistic for survival curves [22]. The RMST is the median survival time of all subjects followed up to time t, obtained from the area under the corresponding Kaplan-Meier curve of the survival function up to *t*. The RMST was estimated at *t* = 24, 60 and 120 months after treatment or after diagnosis [23]. We considered a RMST difference, but not a RMST ratio, as the measure of effect because it may be intuitively understandable to clinicians. Comparisons between RMST adjusted for gender and age were made using an adjusted ANCOVA-type analysis, as proposed by Tian [24–26]. RMST values with 95% confidence intervals were estimated using the ‘survRM2’ package of the R programming language. The significance level adopted was 0.05.

## Abbreviations

GA: gastric adenocarcinoma
GAC: gastric adenocarcinoma coastal
GAM: gastric adenocarcinoma mountainous
HER2: human epidermal receptor 2
HIF: Hypoxia Inducing Factor
SOLCA: Sociedad de Lucha contra el Cáncer
TNM: tumor node metastasis
RMST: restricted median survival time
ΔRMST: restricted median survival time difference
